# Genomic characterization of MDR/XDR-TB in Kazakhstan by a combination of high-throughput methods predominantly shows the ongoing transmission of L2/Beijing 94–32 central Asian/Russian clusters

**DOI:** 10.1186/s12879-019-4201-2

**Published:** 2019-06-24

**Authors:** B. J. Klotoe, S. Kacimi, E. Costa-Conceicão, H. M. Gomes, R. B. Barcellos, S. Panaiotov, D. Haj Slimene, N. Sikhayeva, S. Sengstake, A. R. Schuitema, M. Akhalaia, A. Alenova, E. Zholdybayeva, P. Tarlykov, R. Anthony, G. Refrégier, C. Sola

**Affiliations:** 1 Institute for Integrative Biology of the Cell (I2BC), CEA, CNRS, Univ. Paris‐Sud, Université Paris‐Saclay, 91198 Gif-sur-Yvette cedex, France; 2 Laboratory of Molecular Biology Applied to Mycobacteria, FIOCRUZ, Rio de Janeiro, Brazil; 3 Center of Scientific and Technological Development (CDCT), Secretary of Health of Rio Grande do Sul State (SES/RS), Porto Alegre, Brazil; 4 National Center of Infectious and Parasitic Diseases, Sofia, Bulgaria; 5 Institut Pasteur de Tunisie, Tunis, Tunisie; 6 National Centre for Biotechnology, Astana, Kazakhstan; 7 Royal Tropical Institute (KIT), Amsterdam, The Netherlands; 8 Foundation for Innovative New Diagnostics (FIND), Geneva, Switzerland; 9 National Centre for Tuberculosis Problems, Almaty, Kazakhstan

**Keywords:** Kazakhstan, Tuberculosis, MDR-TB, XDR-TB, Genomics, Public health, Molecular evolution, High-throughput diagnostics methods

## Abstract

**Background:**

Kazakhstan remains a high-burden TB prevalence country with a concomitent high-burden of multi-drug resistant tuberculosis. For this reason, we performed an *in depth* genetic diversity and population structure characterization of *Mycobacterium tuberculosis* complex (MTC) genetic diversity in Kazakhstan with both patient and community benefit.

**Methods:**

A convenience sample of 700 MTC DNA cultures extracts from 630 tuberculosis patients recruited from 12 out of 14 regions in Kazakhstan, between 2010 and 2015, was independently studied by high-throughput hybridization-based methods, TB-SPRINT (59-Plex, *n* = 700), TB-SNPID (50-Plex, *n* = 543). DNA from 391 clinical isolates was successfully typed by two methods. To resolve the population structure of drug-resistant clades in more detail two complementary assays were run on the L2 isolates: an IS*6110*-NTF insertion site typing assay and a *SigE* SNP polymorphism assay.

**Results:**

Strains belonged to L2/Beijing and L4/Euro-American sublineages; L2/Beijing prevalence totaled almost 80%. 50% of all samples were resistant to RIF and to INH., Subtyping showed that: (1) all L2/Beijing were “modern” Beijing and (2) most of these belonged to the previously described 94–32 sublineage (Central Asian/Russian), (3) at least two populations of the Central Asian/Russian sublineages are circulating in Kazakhstan, with different evolutionary dynamics.

**Conclusions:**

For the first time, the global genetic diversity and population structure of *M. tuberculosis* genotypes circulating in Kazakhstan was obtained and compared to previous local studies. Results suggest a region-specific spread of a very limited number of L2/Beijing clonal complexes in Kazakhstan many strongly associated with an MDR phenotype.

**Electronic supplementary material:**

The online version of this article (10.1186/s12879-019-4201-2) contains supplementary material, which is available to authorized users.

## Background

Kazakhstan is a Former Soviet Union country in Northern Central Asia with 18.3 million inhabitants. In 2016 it was one of the 30 countries worldwide with the highest burden of multidrug-resistant tuberculosis (MDR-TB) (www.stat.gov.kz and [1]). Apart from resistance to Rifampin (RIF) and Isoniazid (INH) that defines MDR, resistance to streptomycin (SRM), fluoroquinolones (FLQ) and other second-line injectable drugs (SLID) is common in MTC isolates from Russia and in Former Soviet Union (FSU) republics [1, 2]. Such a phenomenon creates a threat to the effective global control of TB infection. According to a recent World Health Organization (WHO) report, extensively drug-resistant TB (XDR-TB) had by the end of 2016 been reported by 123 countries [1], i.e. in 6 countries more compared to the situation one year earlier [3].

Kazakhstan has established an efficient surveillance system to monitor drug resistance in the past three years, with initially 18 tuberculosis dispensaries (14 regional, 2 urban and 2 zonal), recently increased to 27 [1, 4]. Phenotypic Drug susceptibility Testing (DST) is performed by 22 laboratories and 12 laboratories are reported to be able to run Line Probe Assays (LIPAs, Hain Diagnostics, Germany). Among the 30 high MDR-TB burden countries, 14 had MDR/RR-TB cohorts in 2014 with more than 1000 cases [1]. Among these, Kazakhstan, (together with Myanmar and Viet Nam) reported treatment success of more than 75%. In this context, high-throughput predictive genotyping of drug susceptibility testing could complement and be a faster alternative to phenotypic DST or LIPAs, with high reliability if done in a limited number of certified reference laboratories. As such it could help monitoring as well as targeted DST testing for the bacteriologically confirmed cases (9597 in 2014) [5].

TB-SPRINT and TB-SNPID are Nucleic-Acid PCR-based Amplication Tests (NAAT) that provide simultaneous MTC genotyping subspecies identification and first-line (TB-SPRINT) and first and second-line (TB-SNPID) predictive drug-resistance genotyping (see Table 1 for marker description) [6–11]. These assays use the versatility provided by microsphere hybridization systems that are analyzed either by flow cytometry or by fluorescence imaging using high-throughput analytical devices [12, 13]. TB-SNPID is a multiplex ligation-dependent probe amplification (MLPA) assay whereas TB-SPRINT and TB-EFI rely on direct hybridization and on a dual-oligonucleotide priming (DPO) principle [14, 15].

**Table 1:** Drug-resistance markers analyzed in the two high-throughput microbead-based assays

DR Markers assesed	TB-SPRINT	TB-SNPID
rpoB_176_mut		x
rpoB_516_GAC_wt	x	
rpoB_516_GTC_mut1	x	x
rpoB_516_TAC_mut2	x	
rpoB_522		x
rpoB_526_CAC_wt	x	
rpoB_526_TAC_mut1	x	x
rpoB_526_GAC_mut2	x	x
rpoB_531_TCG_wt	x	x
rpoB_531_TTG_mut1	x	
rpoB_531_TGG_mut2	x	
katG_315_AGC_wt	x	x
katG_315_ACC_mut1	x	
katG_315_AAC_mut2	x	
inhA_-15_C_wt	x	
inhA_-15_T_mut1	x	
inhA_-16_A_wt		
inhA_-16_G_mut1		
inhA_-16_G_wt		
inhA_-8_T_wt		
inhA_-8_A_mut1	x	
gyrA_90-91_GCGTCG_wt		x
gyrA 94_wt_GAC		x
rrs 1401_wt_A		x
rrs1402_wt_C		x
eis_(−10A)_CACAA		x
eis_(−14 T)_TACAG		x
rpsl-43		x
rpsl-88		x
rplC-460		x
Reference	*Gomgnimbou* et al. *2012; Gomgnimbou* et al. *2013*	*Bergval* et al. *2012; Sengstake* et al. *2014*

**Fig. 1:**
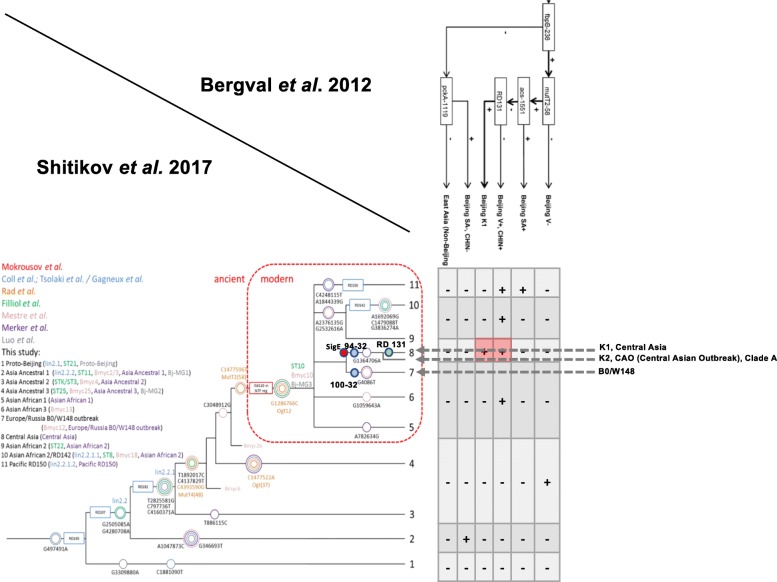
Algorithm of current updated taxonomy of *Mycobacterium tuberculosis* L2/Beijing isolates by TB-SNPID adapted from Shitikov et al. 2017 (11 sublineages based on WGS). The 4 genes used in TB-SNPID are mentioned on top left with SNP variation description: *fbpB, pckA, acs, mutT2* and on RD131 (Niemann et al. 2009, Bergval et al. 2012); TB-SNP-ID naming and classification (top right corner) refers to the geographically named groups based on Niemann et al. 2009 (K1) and on Schürch et al. 2011 (CHIN-, CHIN+, SA-, SA+, V-, V+). The matrix depicts the correspondance between Shitikov’s and previously published taxonomy. Orange boxes are all fbp+. On the tree, all current validated SNP markers are reported with previous other author’s nomenclatures. The *ancient* versus *modern* L2/Beijing nomenclature refers to previous works from I. Mokrousov et al. 2005 based on the presence/absence of IS*6110* copies in the NTF

The main objective of this study was to provide an *in depth* characterization, at the most reasonable cost for public health, of the genetic diversity and population structure of MTC in Kazakhstan, with both a potential patient (personalized treatment) and community (molecular epidemiology) benefit. An accessory goal was to check the congruence between the two methods as a surrogate for their quality: if they are congruent, they are likely to be both reliable. If so, they could represent a less sophisticated-less costly high throughput alternate to Next Generation Sequencing (NGS) for surveillance of MDR-XDR-TB in resource-limited countries. Moreover, the understanding of the complex L2 lineage’s evolutionary history and diversity was recently improved, and a correspondence between numerous authors, diverse genetic markers, and diverse naming has been established. This allows us to use Regions of deletions, SNPs and other markers for L2 epidemic clusters definition (Fig. 1) [6, 16–18]. In Russia, China and FSU republics such as Uzbekistan and Kazakhstan where the prevalence of Beijing is high, methods allowing discrimination within the L2/Beijing are essential to allow MDR-TB evolutionary genomics to be studied [2, 19, 20].

We report in this paper the genotyping results obtained on isolates collected between July 2010–November 2015, i.e. a set of 700 MTC DNAs representative of 630 patients (70 duplicates DNA) that were provided by the National Center for Biotechnology of the Republic of Kazakhstan in Astana, working in collaboration with the National Center for Tuberculosis Problems in Almaty. We compare our results to those obtained previously and assess the dynamics of L2/L4 in Kazakhstan.

## Methods

### Patients, demography

Isolates from a total of 630 patients living in Kazakhstan (435 men, 195 female) were included in the study. Patient data were only available for patients recruited in the Kazakh National Tuberculosis Reference Laboratory. New cases of tuberculosis accounted for 35.6% of the cases (224 of 630) and 64.4% (*n* = 406) were previously treated patients. Among 630 patients with pulmonary tuberculosis 69% (*n* = 435) were men and 23% (*n* = 145) were women, 50 (8%) were unknown. The age of TB patients varied from 16 to 80 years, with an average age of 37 years.

### Ethics

Public health action taken as a result of notification and surveillance is one of the Public Health National Centre for Tuberculosis of Kazakhstan key roles as stated by the Ministry of Health Act and subsequent Government directives which provide a mandate and legislative basis to undertake necessary follow-up. Part of this follow-up is identification of epidemiological and molecular links between cases, helped by the National Center for Biotechnology that provides expertise and links to the Ministry of Research. This study is part of service development carried out under this framework, and as such explicit ethical approval was not mandatory. Moreover patient data are not traceable by any other third part than by the National Centre for Tuberculosis.

### Clinical isolates, TB identification, phenotypic DST

Clinical isolates of MTB were collected from ten regional TB dispensaries (Atyrau, Mangistau, Aktobe, Kostanay, Pavlodar, Semey, Kyzyl-Orda, Zhambyl, Taldykorgan and Almaty) and two prisons run by the State-established committee for the management of the penitentiary system of the Karaganda region and the committee for the management of the penitentiary system of the Akmola region. Isolates were collected from all major regions of Kazakhstan, representing the territory of Kazakhstan. Accordingly, our samples characterize the lineage prevalence of tuberculosis in Kazakhstan as a whole. The high level of sampling from retreated cases however makes it inappropriate to evaluate drug-resistance prevalence in the general population. Clinical isolates of *M. tuberculosis* were also deposited in the Reference Laboratory of the National Center of Phtisiopulmonology for surveillance studies of anti-TB drug resistance in Kazakhstan. After primary isolation on Löwenstein-Jensen (LJ), mycobacteria were subcultured on the same medium in regional laboratories. Species identification as *M. tuberculosis* and drug susceptibility testing were performed at the Reference Laboratory by standard procedures [21]. Rifampicin and isoniazid susceptibility tests were carried out on LJ medium containing 40 μg/L rifampicin, 0.2 mg/L isoniazid, or 1 mg/L isoniazid using the absolute concentration method according to the World Health Organization (WHO) recommendations.

### DNA extraction methods

DNA was extracted by thermolysis. Briefly, colonies were pelleted, culture medium was discarded, and colonies resuspended in TE (10 mM Tris-HCl, 1 mM EDTA, pH 7.0). Samples were heated to 95 °C for 45 min. The suspension was centrifuged at 15,000 g for 1 min to pellet the cell debris. The supernatant containing the DNA was harvested and transfer into a new tube. The stocks were stored at − 20 °C until further use, or diluted at 1:50 into sterile water in a new tube. Seven hundred DNAs were shipped to France and to the Netherlands for further studies in two shipments (June 2015, *n* = 93, August 2015, *n* = 607).

### Multiplexed genotyping methods

#### Tb-sprint

TB-SPRINT typing (59-Plex) is a modular 43-Plex (spoligotyping) plus 16-Plex (first-line drug resistance typing) NAAT developed for Luminex 200®, MagPix® and FlexMap3D® systems (Luminex Corp, Austin, TX) [22]. In this study, two consecutive experiments, firstly spoligotyping, secondly first-line drug-resistance typing were run. Readings were performed on a Luminex 200® analyzer using xMAP® coupled microspheres (Beamedex® SAS, Orsay, France). Second-line drug resistance typing was also tested by the new TB-EFI, a 18-Plex method [23]. Full results are shown in (Additional file 1: Tables S1 and S3 677 spoligotyping results with lineage and sublineage and prevalence percentage table).

#### Tb-SNPID

TB-SNPID was done as previously described [6] using semi-automated data analysis based on a threshold value as published in [8]. Readings were performed on a MagPix® using the MagPlex xTAG® microspheres and reagents provided by MRC Holland (Amsterdam, The Netherlands). DNA samples were first diluted 1:3 in TE containing RNase A (Roche Biochemicals, Pensberg, Germany). Each sample was run in a single well; no samples were repeated. A total of 543 samples were run, however 73 did not pass through the quality check. Hence the final results analysis was performed on 470 samples. Full results are available in (Additional file 1: Table S2).

### NTF-analysis insertion typing of IS*6110* insertion in L2 isolates

A PCR-based agarose gel method was used to analyze the presence/absence of 0–2 copies of IS*6110* in the NTF locus of the L2/Beijing isolates DNAs to discriminate between modern and ancient L2/Beijing [24]. Moreover, a microsphere-based method of this original protocol was also developed and confirmed the gel-based results (results not shown, method to be described elsewhere).

### *SigE* polymorphism analysis

A PCR-RFLP analysis specifically targeting the *sigE* polymorphism that characterize a mutation specific of the 94–32 L2/Beijing cluster (Central Asian/Russian, including the K1 strain from Uzbekistan) was also implemented [18, 25, 26]. The targeted mutation, when present, creates a double band of 38 and 37 bp by creation of a new *AluI* site in codon 38 through a CTG - > CTA mutation.

### Taxonomic assignations (nomenclature) of molecular results; analysis of 94–32 cluster prevalence

We applied the latest whole-genome sequencing-based (WGS) taxonomical assignation in 7 lineages (L1-L7) complemented by sublineages (i.e. L4.3 for LAM) when a correspondence between spoligotyping-based naming and NGS-based naming was found [27–30]. VNTR clones are designated according to [18, 25, 26] and L2/Beijing sublineages designation was done according to [17]. Of note, in this regard, TB-SNPID performs better than spoligotyping by sub-classifying L2/Beijing isolates in six subgroups based on SNPs and regions of deletions (RD105, *mutT2*–58, *fbpB-*238, *pckA-*1119, *acs-*1551, RD131). Conversely spoligotyping allows a finer classification of L4 isolates: at least ten accepted sublineages whereas TB-SNPID assigns only four L4 sublineages (L4.1.1/X, L4.3/LAM, L4.1.2/Haarlem, L4/Euro-American others). Interestingly, TB-SNPID also correctly identifies the previously spoligotyping-misclassified T signatures, now designated as “LAM-RUS” [31, 32].

### GIS systems and maps building

QGIS v3.0 (www.qgis.org, Girona version) was installed on Mas OSX (Apple, Cupertino, CA); Free Kazakhstan shapefiles were downloaded from http://www.diva-gis.org (www.gadm.org, version 2.5, July 2015). Available published results as well as our results were recorded into the required csv file format [33, 34]. Geographical coordinates of main cities in Kazakhstan were downloaded by Google® search and and Latitude and Longitude were added to the csv files. Apple pies charts were built following QGIS user’s manual.

### Statistics

Chi-squared test or Fisher’s exact test were used for all statistical results presented in the study; they were either run using Excel® formulas or using an on-line available calculator (https://biostatgv.sentiweb.fr).

## Results

### Lineage and intra-lineage MTC genomics characterization using TB-SPRINT and TB-SNPID

TB-SPRINT and TB-SNPID were respectively performed in France and in the Netherlands; each location was blinded to the results generated in the other location. Definitive results from both centers were then compared and cross-checked. One hundred percent identification concordance was obtained on the first studied set of 93 samples (cf. Additional file 1: Table S4, columns E and Q) and 99.3% of concordant subspecies identification was found at the end of the study with only two unexplained discrepancies. Intra-species typability was better by spoligotyping 96% typability (23/700 failed) than by TB-SNPID 86.6 typability (73/543 failed). The L2/Beijing represented 79% (536/677 = 79.1% by TB-SPRINT; SIT1 *n* = 529, SIT190 *n* = 5, SIT260 *n* = 1, SIT1674, *n* = 1; and 374/470 = 79,5% using TB-SNPID on an partly independent sample set). The total number of isolates identified as non-L2 by the two methods was around 22% (96/470 = 20.4% by TB-SNPID and 24.2% =164/677 by TB-SPRINT).

The most prevalent L4 sublineage was L4.3/LAM (*n* = 51), that includes the so-called LAM-RUS sublineage (previously T1 or T5-RUS2 in spoligotyping-classification) [31, 32, 35]; the second non-L2 most prevalent lineage was the L4.2.1/URAL genotype [36] (*n* = 39, labeled as “Euro-American Other” by TB-SNPID). Five isolates were labeled as “Haarlem” (L4.1.2) by TB-SNPID. By spoligotyping, twelve spoligotypes not reported in the SITVIT database were found, among which 4 were also identified as bona fide L4 isolates by TB-SNPID (others: ND). At least three of these non reported spoligotyping patterns were likely linked to represent mixed infection.

No L2.1 (proto-Beijing) nor L2.2.2 (Asia Ancestral 1) were found based on *fbpB-*238 results in TB-SNPID. As expected, no L2.2.1.1 (Pacific RD150) was found in Kazakhstan based on *acs-*1551 SNPs. All L2/Beijing identified isolates were further shown to belong to the L2/Modern subgroup (Fig. 1) by the gel-based IS*6110*-NTF method, confirmed by an NTF-IS*6110* microsphere-based assay (results not shown). L2 was further split by TB-SNPID into: Beijing SA+ (*n* = 163 or 34%), Beijing V+/CHIN+/K2 (n = 16 or 3.4%), Beijing (V-) (*n* = 73 or 15.5%), Beijing K1 (*n* = 22 or 4.6%). The correspondence between this designation and the current accepted L2 sublineage nomenclature is shown in Fig. 1, for instance K1 isolates belong to the L2 sublineage 8 from Shitikov et al. [17]. Other L2 epidemic clonal complex are found within these two sublineages [19].

### Genotypic predictive drug-resistance results

Simultaneous precise identification and predictive drug-resistance typing is of great clinical and epidemiological interest. Typeability on drug-resistance conferring mutations was 76% for TB-SPRINT, and 86.3% by TB-SNPID. Among the set of samples run by the two methods (*n* = 391), we detected 257 MDR by TB-SPRINT and 247 by TB-SNPID and 75 fully susceptible isolates by TB-SPRINT and 67 by TB-SNPID. All other isolates showed various resistance genotypes (see Additional file 1: Tables S1, S2, S5). MDR prevalence of the isolates in this study lies at 60% (*n* = 273/470 i.e. 58.1% by TB-SNP-ID; *n* = 344/515 i.e. 66.8% by TB-SPRINT); full susceptibility around 20% (93/240 = 19.9% and 100/515 = 19.4% respectively); monoresistance to INH around 15% (86/470 and 67/515 = 13%) and RIF monoresistance around 2% (18/470 and 4/515). These figures do however not represent actual resistance prevalence as our sampling was strongly biased in favor of retreated cases (64.4% of the total isolates).

Based on TB-SPRINT, 62.3% L2/Beijing isolates (320/421) were MDR whereas only 4.6% non-L2/Beijing isolates were (24/94). The statistical significance of this result is very high (odds ratio = 9.19 IC_95_ [5.39, 16.13], *p* < 2.2 10^− 16^), showing that L2 lineage and MDR-TB are strongly associated in Kazakhstan. Table 2 gives a detailed distribution of resistance patterns according to Lineage and sublineage: Beijing SA+ and V+/CHIN+/K2 were more likely to be MDR than Beijing K1 and Beijing −/−/−/− (*p*-value = 1.38 .10^− 7^, Fisher’s exact Test, Additional file 1: Table S8). L4.3 showed a non-significant trend to be more likely MDR as compared to L4.1.2/Haarlem and L4 -EA-other.

**Table 2:** Detailed combined Identification and predictive drug-resistance results obtained by TB-SNPID on first-line drugs

	Total	S	monoR-INH	monoR-RIF	MDR	EMB
Euro-American Other	39	25	9	1	4	2
LAM (L4.3)	51	12	10	9	20	1
Haarlem (L4.1.2)	5	3	1	0	1	1
L2/Beijing/K1	22	4	6	2	10	7
L2/Beijing/V+/CHIN+/K2	116	2	15	2	97	92
L2/Beijing/SA+	163	5	35	1	122	115
L2/Beijing/−/−/−/	73	12	10	3	18	10
/−/−/−/	1	0	0	0	1	0

RIF resistance associated mutations occurred mostly in *rpoB* codon 531 (60%; 273/470 and 320/515). Other RIF resistance associated mutations concerned positions 522 (around 3%; 13/470 by TB-SNPID; not assessed by TB-SPRINT) and 526 (around 2.7%; 11/470 and 14/515) and 516-GTC (around 1%; 4/515 by TB-SPRINT, not assessed by TB-SNPID). Some double mutations were detected (14/515 samples analyzed by TB-SPRINT; Additional file 1: Table S1). No mutations were detected in *rpoB* at codon position 176.

Regarding INH resistance associated mutations, the *katG* 315 S > T mutation was found in more than 70% of the isolates (348/470 and 385/515), and *inh* − 15 mutation in around 5% of the samples (34/470 and 5/515). Some double mutations were detected (15/515 samples analyzed by TB-SPRINT; Additional file 1: Table S1).

EMB resistance as associated mutations in *embB* 306 were found in half of the samples (228/470). Second-line resistance associated mutations in relation to SLID and FQ resistance were detected in decreasing order: the *eis*G-10A mutation (72/470), *rrs*1401 (34/470), *gyrA*94 mutations (30/470), *gyrA*90 mutations (22/470), *eisG-14 T* (7/470; Additional file 1: Table S2).

### *In depth* georeferenced characterization of L2/Beijing and first attempt to characterize the evolutionary dynamics of L2/L4 clones circulating in Kazakhstan

To get a deeper insight on L2 clones circulating in Kazakhstan, we further undertook complementary genomic analysis on a total of *n* = 356 DNAs (seven L4 as controls, three “failed” by TB-SNPID, and 346 L2/Beijing isolates). We used the recently described *SigE* SNP characterization method which was shown to be specific of the 94–32 Central Asian/Russian L2/Beijing cluster [18, 25, 26]. We obtained 314 mutants out of 346 L2-isolates characterized (90%), which demonstrates the presence of a limited number of epidemic clones belonging to the Beijing 94–32 cluster. We show that at least two different L2/Beijing epidemiologically linked clusters were circulating in the prison in Stepnogorsk, with at least respectively 20 cases (94–32 cluster) and 8 cases (L2/Beijing second cluster), whereas in Aktobe, a single drug-sensitive L2 clone was circulating during the studied time-frame. This clonal complex is encompassed in the TB-SNPID L2/Beijing/−/−/−/ label (Additional file 1: Table S7).

**Fig. 2:**
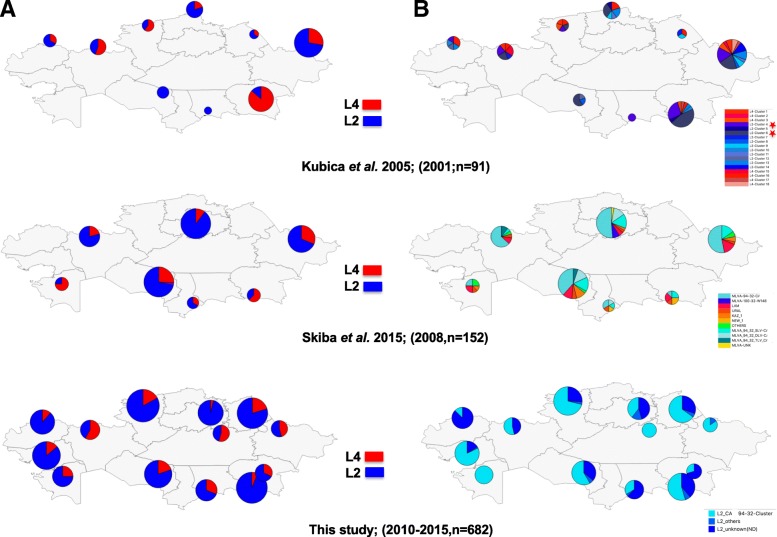
Six QGIS® built genetic maps of Kazakhstan showing *Mycobacterium tuberculosis* based on samples recruited respectively in 2001 (*n* = 91), 2008 (*n* = 152) and 2014–2015 (*n* = 632) **a**: from top to bottom: The L2/Beijing (Blue) and L4-Euro-American (Red) relative prevalence based on three different studies published in 2005 (Kubica et al.), 2015 (Skiba et al.) and in this study suggests an increase of prevalence of L2 relatively to L4. **b**: from top to bottom: the assessment of epidemiological clusters, done by different methods (IS*6110*-RFLP by Kubica et al. 2005, MLVA by Skiba et al. 2005; a SNP-based (SigE) specific for the 94–32 cluster found in this study, shows a dynamic increase towards the prevalence of a very limited number of variants of the 94–32 cluster. Red stars identifies majors IS*6110*-RFLP clusters 4 and 6, identified in the 2001 study

We then performed a thorough georeferencing using a geographic information system (GIS), synthesizing TB outbreak information of a 15-year period (2001 to 2015). Results are shown in Fig. 2. In the 2005 study published by T. Kubica et al. on 150 patients recruited in 2001 in 9 provinces of Kazakhstan, only 91 isolates could be georeferenced (Fig. 2a). L4 looks quite prevalent at that time compared to L2/Beijing (Fig. 2, left, top), especially in the south-east of the country (Almaty). In the 2015 study published by Y. Skiba et al. on a total of 152 patients recruited in 2008 in 9 provinces of Kazakhstan, a trend towards an increase of the L2/Beijing prevalence can be noticed, with a still high prevalence of L4 in Almaty (Fig. 2a, central). However in our study, performed on a sample of 632 patients in 13 regions, the prevalence of the blue lineage (L2/Beijing) outnumbers the L4, at least in some regions (Fig. 2a, bottom), including Almaty. A statistical analysis demonstrates that in Almaty, the proportion between L2 and L4 is not homogeneous (Chi2 = 52,5; *p* < 0.001; DF = 2); there is a recent higher prevalence of L2 compared to L4, the same is true in Kostanay (however in that setting, only two time points were available, in 2001 and in 2010–2015). No other statistically significant results were obtained regarding tuberculosis lineage distribution evolution. Of note in the Kyzyl-Orda region where L4 seems to increase between the 2008 and 2010–2015 period, the trend was not statistically significant (see Additional file 1: Table S7).

In Fig. 2b**,** we retrospectively analyzed the evolution of the prevalence of the L2/Beijing epidemic clones in Kazakhstan, as they had been characterized by different methods: by IS*6110*-RFLP in the first study, by 24 VNTR typing in the second study, and by combination of various markers (including RD131 and *SigE* SNP polymorphism) in our study. Figure 2b shows that a limited number of clones (likely IS*6110*-RFLP clusters 4 and 6 in the 2005 article and Beijing 94–32 cluster in the 2015 article) were already circulating in Kazakhstan between 2001 and 2008 (Fig. 2b, top and central). A further comparison of the IS*6110*-RFLP K1-K2 patterns obtained in a study run in Uzbekistan (isolates obtained during 2001–2004) showed that some IS*6110*-RFLP clusters found in Kazkakhstan were highly similar if not identical to the IS*6110*-RFLP patterns described simultaneously in the Uzbekistan study (Additional file 2: Figure S3) [37]. We performed preliminary typing on 8 VNTR on a limited set of L2/Beijing DNA and obtained results that were similar to those obtained by Skiba et al. 2015 confirming the high prevalence of the 94–32 cluster (results not shown). Finally, the last map of Fig. 2b (bottom), provides the *SigE* polymorphic results, and shows a clear geographic picture of L2/Beijing 94–32 MDR and XDR transmission that suggests an increase of the transmission of the 94–32 L2/Beijing Central Asian/Russian clusters in Kazakhstan.

## Discussion

In this study, we first performed an *in depth* genomic characterization of MTC isolates circulating in Kazakhstan over an extended sampling time frame (5 years) using a combination of high-throughput NAATs, with the global aim of simultaneously improving MDR-TB surveillance as well as providing the possibility to more accurately personalize TB treatment; complementary genomic characterization using IS*6110* copy number characterization in the NTF region demonstrates the exclusive presence of Modern L2/Beijing strains in Kazakhstan. Further *SigE* polymorphism analysis, together with georeferencing, gives a deep dynamic picture of the 2010–2015 TB outbreak in this country; we suggest that the L2/Beijing outbreak in Kazakhstan is essentially linked to the increased spread of 94–32 L2/Beijing clusters, with regional specificities such as in Almaty, Aktobe and to a lesser extent Kostanay, that may deserve further studies using NGS, in order to put these results into a wider phylogeographic context and to compare with the situation in the neighboring Uzbekistan [2] and Kyrgyzstan [38]. A new whole-genome based study that could characterize further the isolates identified on the basis of their SNPs from the Central Asian/Russian and CAO outbreak clones would provide a deeper insight into the genetics of these strains of TB in Kazakhstan [26].

Secondly we demonstrated the use of TB-SNPID, a 50-Plex assay that combines species, subspecies and sometimes (for targeted genotypes) highly precise clonal identification simultaneously to drug-resistance gene markers; and we used a new spoligotyping - first-line drug typing direct hybridization assay [6, 8, 22, 39–42]. When independently run on crude DNA extracts these methods proved to be highly congruent, only partially redundant and quite complementary on a set 391 clinical samples that were blindly typed by both methods.

This study is the first of its kind to characterize as precisely, at a national and population level, a large set of TB isolates by genomic non-NGS methods.

### Advantages, inconvenience of and discrepancies between the two NAATs

In this study, TB-SPRINT and TB-SNPID showed some discrepancies regarding the presence of mutations conferring RIF or INH resistance. Part were intrinsically linked to differences in assay design as shown in Table 1. A thorough discrepancy analysis was undertaken on the first set of 93 isolates (Additional file 1: Table S4). This set of samples showed that only one true and unexplained discordance (red color in Additional file 1: Table S4) was found between the two NAATs. The second discrepancy analysis on all 391 results detected 34 discrepancices between the two NAATs (91.3% of congruence). We did not re-run discrepant results because of lack of budget. Full results are available in Additional file 1: Tables S5 and S6.

Phenotypic DST MDR status was almost always correlated with the NAAT results (*n* = 9 discrepancies). For six cases, there was a partial agreement on INH resistance, for three others on RIF resistance. Our assays showed altogether limited discrepancies between phenotypic and genotypic DST, in relation to their simple design compared to the complexity of drug resistance emergence and adaptation mechanisms [43]. Discrepancies observed in this study between phenotypic and genotypic DST may be due to errors in phenotypic DST or in genotypic DST since budget was limited to rerun some samples. Infections with mixed resistance genotypes in the patient population may account for another proportion of these discrepancies [44]. Even if phenotypic DST is often still considered as the reference standard, there are inherent difficulties linked to this method, that require quality assurance procedures to track potential errors [45]. In United Kingdom, the switch from phenotypic to genotypic DST is a new reality [46].

Both TB-SPRINT and TB-SNPID methods have advantages and inconveniences: one advantage of TB-SNPID is the “one tube/one run” concept on the MagPix® whereas TB-SPRINT is more suited to Luminex 200® or FlexMap 3D® users. Compared to WGS, TB-SPRINT and TB-SNPID are targeted assays that detect what they are designed to, i.e. a remaining number of samples will always need to be sequenced in particular to discover new mutation responsible of drug resistance. This provides the possibility to adapt these assays to detect specific genotypes. In contrast, WGS is a universal genotyping approach [43]. We recently reported second-line drug resistance assays in Kazakhstan using the 18-Plex “TB-EFI” that targets mutations in *eis, gyrA, rrs*, *embB* [23]. Such an assay can be run for a very limited cost in settings where MDR and preXDR-TB is present. Other assays such as the Deeplex® from Genoscreen (Lille, France) are other alternative solution to NGS [47]. Predictive WGS-based bioinformatical pipelines represent an important step forward for TB drug-resistance containment and these methods will progressively spread even if they remain quite expensive (200 US$ per isolate) [48] and considering that bioinformatical pipelines are in evolution [49]. Reagent costs are 20 Euros/sample for TB-SNPID and 12 euros/sample for TB-SPRINT. A raw economic analysis suggest a doubling cost to produce final results using european standard labor costs; these costs are lower in emerging economies. Hence, our assays are cheaper and easier to implement on large numbers of samples than NGS studies for any national, population-based, drug-resistance evaluation study.

### Main circulating sublineages in Kazakhstan and MDR transmission risk

Using both general markers provided by the two NAATs and more specialized markers (RD131 in TB-SNPID, *SigE* run independently) we identified that main circulating clones belong to the L2-Beijing Central Asian/Russian 94–32 sublineage. Further characterization could be performed using the 24 specific SNPs identified by the recent in-depth WGS-based analysis of L2/Beijing 94–32 cluster [18].

We also showed that some but not all of these 94–32 clones were associated with an MDR genotype: the K1 clone was in contrast linked to susceptibility [37]. We now have a better picture of drug-resistance mutations, and of L2 and non L2 sublineages circulating in Kazakhstan.

### Surveillance of L2/Beijing MDRs

The L2 taxonomy still needs reconciliation between “ancient” knowledge on IS*6110*-RFLP, VNTR, RDs, and “modern” WGS acquired results to achieve a unified, deeper, and consensus taxonomical definition of L2 epidemic clones [17, 26]. In addition to purely phylogenetic markers, other polymorphic markers should be investigated to better understand the epidemic success of various clones. Markers of particular interest are undoubtedly adaptive compensatory mutations such as those acting on *rpoA* and *rpoC* genes that lower or eliminate the fitness-cost of the drug resistance mutation [19]. An improved global and local knowledge of drug-use history is also of paramount importance to understand the chronology of drug mutation emergence [2, 43]. Looking for epistasis between SNPs, could also be of added value to understand why the 94–32 cluster has been so successful [50].

### TB surveillance in high-burden countries

Achieving improved global tuberculosis control and patient treatment simultaneously is now feasible thanks to WGS or high-throughput SNPs assays. Technology spreading by training should create favorable conditions for sustained laboratory and bioinformatics capacity building or strengthening, and could further be applied to other infectious threats [51, 52]. Genomic assays allow clinical microbiology to change from targeted to nontargeted investigations [53]. However one important issue is the trend towards closed and expensive diagnostics systems development by industrials, that can if not handled carefully contribute to de-skilling and less democratization in health systems. This can generate inequalities due to unaffordable and unsustainable costs as well as creating new local perceptions in populations concerning *global health* issues if simultaneously linked to austerity policies [54, 55]. Given the strongly regionalized phylogeographical structure of the TB outbreaks, a “*one-size fits all*” strategy for MDR-and XDR-TB control may not be the optimal solution; as demonstrated in Swaziland, the prevalence of rare drug resistance mutations such as the *rpoB* I491F, may result in missed MDR-TB cases and inadequate TB treatment and infection control [56]. Global economic issues also remain with respect to sustainability and long-term routine work for WGS-based assays in emerging economies, even if lot of resources are spent to train bioinformaticians [57]. That is the reason why, in the post-genomic era, other locally developed less sophisticated assays, based on and complementary with NGS results, may become important in resource-limited countries [18, 58–60]. Furthermore, the availability of large amounts of WGS data will allow the precise election of genotypes to be screened. They would in turn help to develop second-generation assays that could rapidly be applied to very large number of samples.

## Conclusions

In brief, this study demonstrates that two non-NGS information-rich genomic assays could satisfactorily be run independently and successfully, with minimal costs, to produce information-rich public health datasets for patient and community benefit. Our results also suggest the spread of a limited number of L2/Beijing clusters during the 2010–2015 period, with geographic variations and different drug-susceptible/drug-resistant outbreaks histories.

## Additional files

Additional file 1: **Table S1.** detailed TB-SPRINT results on 700 MTC DNA samples from Kazakhstan (*n* = 677 spoligotypes including 3 potential “mixed infection”, 23 failed). **Table S2.** detailed TB-SNPID results on 470 MTC DNA samples from Kazakhstan (*n* = 543 analysed, 73 failed). **Table S3.** Lineage and Sublineage assignation of Spoligotyping results (n = 677). **Table S4.** All isolates (*n* = 93) of “set1” (i.e. chronologically first set) typed by two methods: TB-SPRINT and TB-SNPID. **Table S5.** Detailed results for *n* = 391 samples run both by TB-SPRINT and TB-SNPID. **Table S6.** Analysis of discrepant drug-resistance results between TB-SPRINT and TB-SNPID on the 391 set of isolates. **Table S7.** congruence between SigE and RD131 (TB-SNPID) results in Aktobe suggests the presence of a single DST L2/Beijing 94–32 clone. **Table S8.** Chi2 square statistics on L2/L4 prevalence in some regions of Kazakhstan. (XLSX 318 kb)

Additional file 2: **Figure S3.** Comparison between IS*6110*-RFLP patterns obtained in Kazakhstan (2001) and Uzbekistan. (2001–2004). (PDF 1710 kb)

## Data Availability

All raw data are released in the Additional file 1: Tables S1-S8. All material will be made available upon request to the corresponding author.

## Abbreviations

AMK: Amikacin
DPO: Dual-Priming Oligonucleotide
DST: Drug-susceptibility testing
EMB: Ethambutol
FLQ: Fluoroquinolones
INH: Isoniazid
KAN: Kanamycin
MDR-TB: Multidrug-resistant tuberculosis
MLPA: Multiple ligation-mediated probe dependent amplification assay
MTC: *Mycobacterium tuberculosis* complex
NAAT: Nucleic acids amplification-based tests
RIF: Rifampin
TB: Tuberculosis
TB-EFI: high-throughput Luminex-based Ethambutol, Fluoroquinolones, second-line Injectable drugs drug-resistance SNPs assay
TB-RINT: high-throughput Luminex-based, first-line drug-resistance SNPs assay (Rifampicin, Isoniazid)
TB-SNPID: high-throughput Luminex-based MLPA typing and identification SNP-based assay
TB-SPOL: high-throughput Luminex-based spoligotyping assay
TB-SPRINT: high-throughput Luminex-based, combined spoligotyping and first-line drug-resistance SNPs assay (Rifampicin, Isoniazid)
SLID: Second-line injectable drugs
WGS: Whole-Genome Sequencing
XDR-TB: extremely-drug-resistant tuberculosis
