# Supplementary material for: Genomic characterization of MDR/XDR-TB in Kazakhstan by a combination of high-throughput methods predominantly shows the ongoing transmission of L2/Beijing 94–32 central Asian/Russian clusters
Source: BMC Infect Dis. 2019 Jun 24;19:553. doi: 10.1186/s12879-019-4201-2 (PMC6592005; doi:10.1186/s12879-019-4201-2)
Supplement: Supplementary file 2 — Figure S3. Comparison between IS6110-RFLP patterns obtained in Kazakhstan (2001) and Uzbekistan. (2001–2004). (PDF 1710 kb) [file 12879_2019_4201_MOESM2_ESM.pdf]

Supplementary Figure 3: IS*6110*-RFLP patterns obtained in Kazakhstan (2001) and Uzbekistan. (2001-2004)

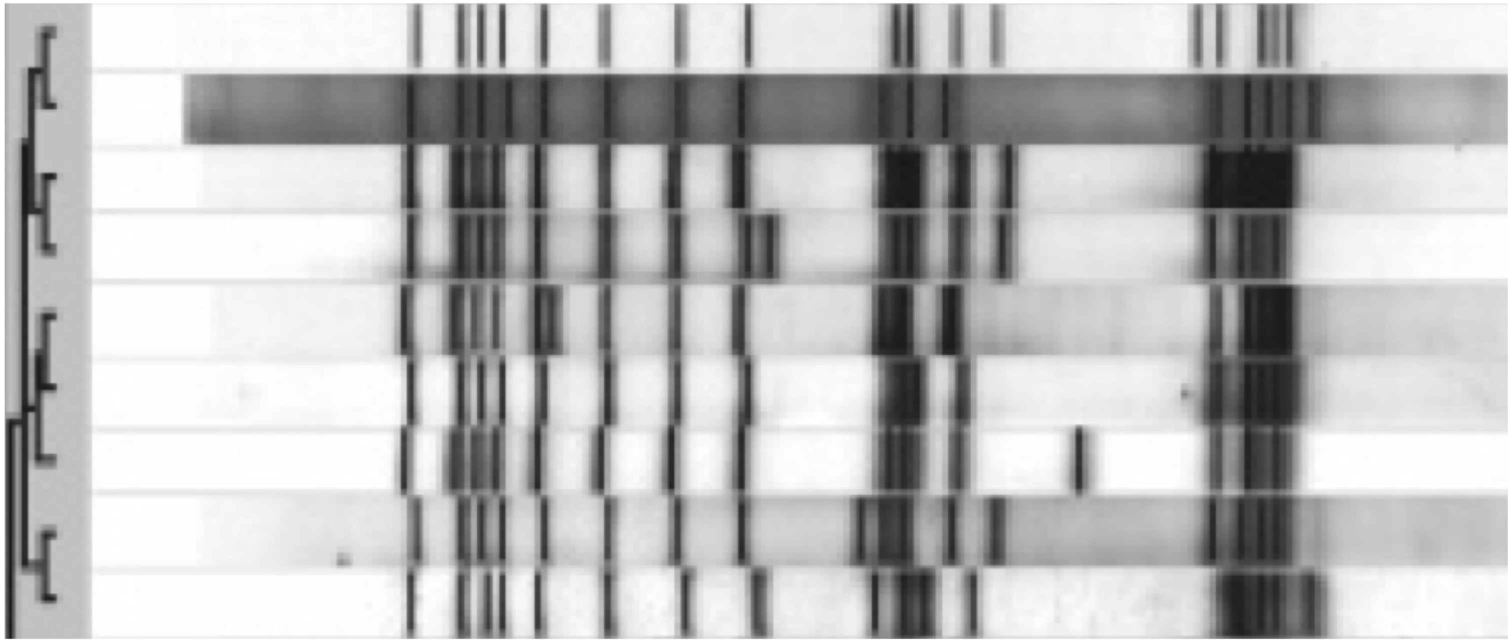

extracted from ***Kubica et al.*** *2005 Figure 3 (Table : correspondance with cluster 4 or 6)*

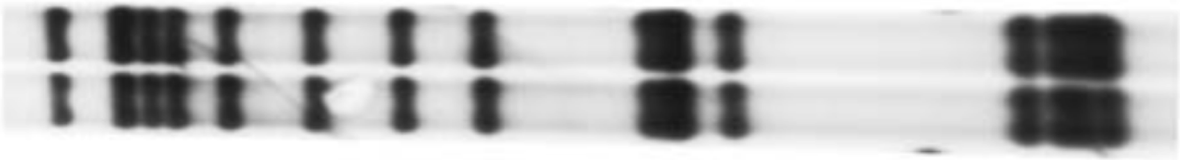

extracted from ***Niemann et al.*** *2009*
